# A Case of Familial Mediterranean Fever with Extensive Lymphadenopathy and Complex Heterozygous Genotype Presenting in the Fourth Decade

**DOI:** 10.1155/2018/9670801

**Published:** 2018-04-01

**Authors:** Jawad Al-Khafaji, Fran Ganz-Lord, Venkata Rajesh Konjeti, Aaron D. Viny

**Affiliations:** ^1^Department of Medicine, Virginia Commonwealth University Health System, Richmond, VA 23298, USA; ^2^CareMount Medical, Chappaqua, NY 10514, USA; ^3^Human Oncology & Pathogenesis Program, Memorial Sloan Kettering Cancer Center, New York, NY 10065, USA

## Abstract

Familial Mediterranean fever (FMF) is an inherited disease caused by loss of function mutations in the *MEFV* gene encoding pyrin, a negative regulator of interleukin-1. The disease is characterized by recurrent fever and self-limited attacks of joint, chest, and abdominal pain but lymphadenopathy is an infrequent manifestation. While mesenteric lymphadenopathy has been described in several cases in the literature; hilar, paratracheal, axillary, pelvic, and retroperitoneal lymphadenopathy are extremely rare and have been reported separately in very few individuals. In this report, we present a patient with late-onset FMF with extensive lymphadenopathy in all of the aforementioned anatomic regions. Genetic analysis identified three heterozygous pyrin mutations in a patient with no affected family members. Genetic investigation of the patient's mother identified a novel carrier haplotype E148Q/P369S. The proband also inherited the previously described and rare A744S mutation previously not thought to be a disease-defining lesion. This unique compound heterozygous genotype resulted in a novel genotype-phenotype association producing an atypical clinical presentation of FMF that fits within the pattern of several case reports of late-onset disease with respect to clinical course and therapeutic response.

## 1. Introduction

Familial Mediterranean fever (FMF) is an inherited autonomous inflammatory disease predominantly affecting people of Mediterranean descent, including Sephardic Jews, Turks, Armenians, and Arabs [1]. The disease is hallmarked by three clinical sequelae [1–3]: (1) self-limited, recurrent episodes of fever (lasting 2–4 days), (2) inflammation of the peritoneal, pleural, pericardial, and synovial membranes and skin, leading to chest and abdominal pain, arthralgias, and erysipelas-like erythema, and (3) systemic AA amyloidosis that leads to nephropathy if left untreated. About 60% of patients with FMF experience their first attack before the age of 10, 90% before reaching 20 years, and nearly all present before age 40 [1, 4]. Late-onset FMF is defined as diagnosis above 40 years of age, which is a rare occurrence, affecting about only 0.5% of FMF patients [4].

The disease has been linked to mutations within the gene *MEFV* on chromosome 16 which encodes pyrin, a protein found almost exclusively in mature granulocytes and is a regulator of interleukin-1- (IL-1-) mediated inflammation [5]. Loss-of-function mutations within the pyrin/marenostrin protein complex are believed to cause dysregulation of IL-1 inflammatory response either by a functional mutation in pyrin or increased sensitivity of pyrin to cleavage by caspace-1 [6, 7]. Originally discovered serendipitously, colchicine has become the mainstay of therapy, ameliorating both the inflammatory response as well as resultant amyloid deposition [8]. While the precise mechanism remains incompletely understood, it is likely that colchicine exerts its effect through impairing inflammatory cell activity through conformational changes to microtubules, and new evidence also implicates transcriptional suppression of inflammatory proteins [9, 10].

The genetic basis for the disease has been well described in the last two decades. Founder mutations have been identified at amino acid positions 680, 694, and 726, all within exon 10, leading to alterations of the C-terminus and comprise the majority (86–93%) of cases. Subsequently, several nonfounder mutations have been found in exons 2, 5, and 10 within well-described haplotypes [11].

In this report, we present a case of a late-onset FMF (age 42 years) with a unique phenotype manifesting with retroperitoneal paraaortic, paratracheal, hilar, pelvic, and axillary lymphadenopathy in the setting of a complex heterozygous genotype implicating A744S as a new disease modifying locus.

## 2. Case Presentation

A 42-year-old man of Armenian descent was admitted to the internal medicine service complaining of intermittent fever and night sweats for one month. The fevers continued for >36 hours, peaked at 40°C, and were associated with diffuse myalgias, rigors, and diaphoresis. He also reported drenching night sweats and a 20 lb. weight loss over the previous month. He does not smoke and drinks alcohol sparingly. His past medical history and family history were unremarkable. His review of systems was otherwise unremarkable. He is married with two children and works at a petroleum tank farm with exposure to benzenes and industrial solvents. His physical exam upon admission was significant for fever of 39.1°C, marked diaphoresis, tenderness to palpation in the lower extremities bilaterally, blanching maculopapular rash on the legs bilaterally and left forearm, and subcentimeter mobile, nontender inguinal lymph nodes bilaterally. Myalgias and fevers continued throughout the hospital stay. His myalgias were only moderately relieved by the aggressive use of opiates and NSAIDs. The patient continued to complain of the same symptoms throughout his hospital stay during which he was evaluated for fever of unknown origin.

Laboratory evaluation was significant for ESR 66 mm/hr, C-reactive protein 10.79 mg/L, creatinine kinase 34 U/L, and lactate dehydrogenase 117 U/L. His complete blood count, liver, kidney and thyroid function tests were all within normal limits, as were his serum electrolytes, iron studies, uric acid, amylase, proteins, and immunoglobulins. Serial blood, urine, and sputum cultures, including mycobacterium, were negative. Serologic testing was negative for HIV, EBV, CMV, HBV, HCV, and Syphilis. A PPD was also negative. His EKG, chest X-ray, and abdominal sonography were normal. Testing for the following autoimmune factors was negative: ANA, anti-dsDNA, RF, anti-smith, anti-RNP, anti-SSA, anti-SSB, and CCP.

A malignancy work-up included bone marrow examination. Core biopsy, aspirate, flow cytometry, molecular pathology, immunohistochemistry, and cultures (for tuberculosis, bacteria, and fungi) were all unrevealing. Flow cytometry of peripheral blood was normal, no atypical cells were identified by peripheral smear, and there was no M-spike detected in serum or urine. Whole body PET/CT scan showed hypermetabolic retroperitoneal enlarged lymph nodes in the left paraaortic region at the level of the renal vessels (largest 1.7 × 2.5 cm) and minimally active lymph nodes in the following regions: right axillary region (1.8 cm), right lower paratracheal region (1.5 cm), right upper lung (8 mm), retrocaval, common iliac, internal iliac and obturator regions bilaterally and left external iliac (all subcentimeter), and the inguinal region bilaterally (largest 1.2 cm).

**Figure 1:**
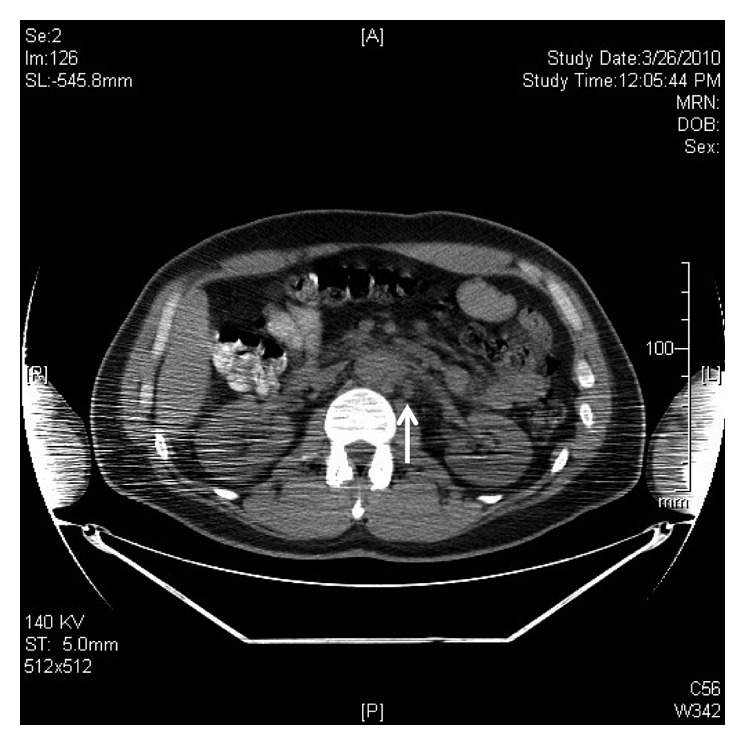
CT image of periaortic lymphadenopathy.

CT-guided aspiration biopsy of the retroperitoneal periaortic lymph node (Figure 1) showed atypical lymphoid proliferation of small lymphocytes, which were polyclonal by flow cytometric analysis. Surgical consultation was requested for an excisional biopsy of the periaortic lymph node; however, this was deferred due to unacceptable risk related to anatomical location of the node.

The patient was given the presumptive diagnosis of a low-grade lymphoproliferative disorder and in addition to careful expectant management, an empiric trial of prednisone was started despite the risk of losing diagnostic lymph node architecture. The patient was discharged and had outpatient follow-up one week later. Over the next several weeks, the patient noted a subjective improvement in his symptoms. However, attempts to taper the prednisone dose resulted in a relapse of fevers and myalgias.

Although very atypical in presentation, the diagnosis of familial Mediterranean fever was considered given the robust response to steroids and his Armenian descent. Genetic studies were performed and an empiric trial of colchicine 0.6 mg daily was also initiated. The patient reported a near immediate relief of symptoms and was then able to be tapered off of prednisone completely. One month later, the *MEFV* genetic results were returned, which confirmed the diagnosis. His genetic tests were significant for a complex heterozygous genotype with the three mutations: E148Q, P369S, and A744S. The patient's mother was also genotyped to determine allelic distribution and risk of inheritance. The patient's mother's test was significant for heterozygosity at the A744S locus. The patient's father was not willing to undergo genetic counseling or testing but denies any symptoms that might be attributed to FMF. Thus, the father's presumed haplotype E148Q/P369S is likely not sufficient to produce any symptoms or clinical phenotype.

**Figure 2:**
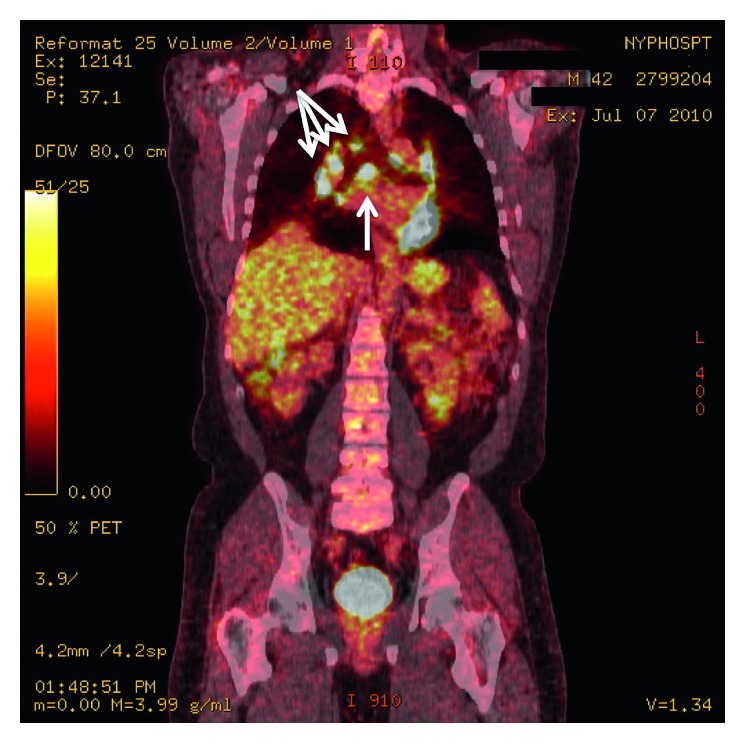
Positron emission tomography imaging of mediastinal lymphadenopathy.

Three months later, a follow-up PET scan was performed to evaluate the patient's previously identified lymphadenopathy (Figure 2). The study revealed near resolution previous paraaortic lymph node and resolved axillary and pelvic lymphadenopathy. However, new extensive hypermetabolic mediastinal and hilar lymphadenopathy were identified, revealing new right upper paratracheal node (1.7 cm), enlarging right lower paratracheal node (1.6 cm), subcarinal node (2.7 cm), and bilateral hypermetabolic hilar lymphadenopathy. At this point, several core needle biopsies of the subcarinal and peritracheal lymph nodes were performed under CT-guidance. Pathologic examination showed nonspecific reactive heterogeneous population of lymphocytes and was negative for granulomatous and amyloidal disease. Molecular pathology, immunohistochemistry, and flow cytometry were unremarkable.

Despite these findings, the patient continued to do well and remained symptom-free on colchicine.

## 3. Discussion

In this report, we describe a unique presentation of an Armenian man with familial Mediterranean fever with extensive lymphadenopathy in the hilum, mesentery, and retroperitoneal regions with a novel complex heterozygous genotype. His genetics implicate A744S as a phenotype defining mutation in conjunction with the E148Q/P369S haplotype.

*MEFV* gene defects in FMF have been well described with mutations clustering at the C-terminus and presumed to affect interactions with regulatory molecules such as 14-3-3 [12]. Other *MEFV* gene mutations found to correlate with disease cluster in the splice junction between exon 1–3 [13, 14]. More than 317 mutations have been identified [15]. The most frequent of which include M694V, M694I, M680I, and V726A [12]. These mutations are associated with a younger age of onset, a variable response to colchicine, and the complications of amyloidosis and renal failure [11, 16, 17]. The distribution, prevalence, and penetrance of FMF mutations seem to have implications in disease severity and age of onset. Although disease most often presents before 20 years of age, onset of the disease after the fourth decade has been reported in only 0.5% of FMF patient population and most had milder disease [4, 18–21]. These patients uniformly had absence of the prototypical M694V mutation, and a favorable response to colchicine was noted in all patients with late-onset FMF compared with other patient populations of younger ages [4]. This suggests that the specific genotype-phenotype association in FMF has a spectrum likely reflecting the capacity of each mutation to maintain varying functional capacity. The heterozygous nature of these mutations predicts a dominant negative effect, with one wild type copy of *MEFV* unable to suppress IL-1 sufficiently.

Therefore, a compound heterozygous genotype with two unaffected parents, as seen in this patient, may reflect limited functional effects of E148Q/P369S and A744S in isolation, but sufficiently reduced function in combination. The former two mutations have been well studied and have been linked to a milder disease phenotype but independently do not cause a disease phenotype [4, 20, 22, 23]. The third mutation is a rare mutation described in the literature but is by itself not particularly associated with any serious disease manifestations [24]. This patient presented with FMF after age 40 and had extensive lymph node involvement. Both of these are rare features but may reflect the slow smoldering nature of this particular genotype, permissive of nodal disease.

When present, lymphadenopathy is most commonly found in the abdomen involving primarily the mesenteric region [25–29]. Retroperitoneal lymphadenopathy has only been reported in 4 patients with FMF [25–28]. Peripheral lymphadenopathy has been reported in only two cases [25, 30]. Paratracheal and hilar lymphadenopathy have each been reported in only one case report [25]. These FMF patient cases with lymphadenopathy that are reported in the literature are summarized in Table 1. When reported, biopsy of these lymph nodes uniformly showed nonspecific lymphoid hyperplasia [25, 26, 30, 31]. In this patient, biopsies of paraaortic, hilar, and paratracheal lymph nodes also showed nonspecific reactive lymphocytes, without infectious or malignant stigmata, and are similar to what has been described elsewhere [25, 26, 30, 31]. The significance of lymphadenopathy in FMF is not well understood, and the clinical significance of their locations is also yet to be studied. Whether they correlate to the disease process (i.e., evolution, severity, and prognosis) is unclear.

This patient has multiple rare findings for FMF. His extensive lymphadenopathy adds to the extremely rare cases reported in the literature of hilar, para-tracheal lymphadenopathy, axillary, and retroperitoneal lymphadenopathy. The age of onset of this patient's FMF illness also adds to the very few cases of late-onset FMF patients. Furthermore, his remarkable complex heterozygous genotype has two-fold impact. First, it establishes a functional consequence of A744S, a rare *MEFV* mutation previously not linked to disease and second, it strengthens the dogma of nonfounder pyrin mutations producing a distinct clinical and prognostic disease.

## Figures and Tables

**Table 1:** Review of literature of lymphadenopathy in familial Mediterranean fever.

Anatomical region	Number of cases	Reference
Abdominal	13	[25, 27–29, 31, 32]
Retroperitoneal^†^	5	[25–28]
Thoracic (hilar and paratracheal)^†^	2	[25]
Peripheral (cervical and axillary)	2	[25, 30]
Pelvic/inguinal^†^	0	—

^†^Anatomic region where lymphadenopathy was identified in the current report.
